# Bacteriuria Among Type 2 Diabetes Mellitus Patients Attending Ejisu Government Hospital in the Ashanti Region, Ghana

**DOI:** 10.1155/2024/1120083

**Published:** 2024-10-30

**Authors:** Constancia S. Dansoa, Nicholas Y. Anaba, Richard T. Zangine, Christine Kodji, Frank A. Bonsu, Gideon O. Abbiw, Isaac Acheampong, Solomon Wireko, Seth A. Domfeh

**Affiliations:** ^1^Department of Biochemistry and Biotechnology, Faculty of Biosciences, Kwame Nkrumah University of Science and Technology, Kumasi, Ghana; ^2^Clinical Analysis Laboratory, Department of Biochemistry and Biotechnology, Faculty of Biosciences, Kwame Nkrumah University of Science and Technology, Kumasi, Ghana; ^3^Department of Medical Diagnostics, Faculty of Allied Health Sciences, Kwame Nkrumah University of Science and Technology, Kumasi, Ghana; ^4^Department of Laboratory Technology, Faculty of Health Sciences, Kumasi Technical University, Kumasi, Ghana

**Keywords:** antibiotic resistance, bacteriuria, Type 2 diabetes mellitus, urinary tract infection

## Abstract

Type 2 diabetes mellitus (T2DM) patients are at increased risk of infections, such as malignant otitis externa and rhinocerebral mucormycosis, with the urinary tract being the most affected (for example, emphysematous pyelonephritis commonly caused by *Escherichia coli*). Hence, this study assessed the prevalence of bacteriuria and antibiogram patterns of bacteria isolates among T2DM patients visiting the Ejisu Government Hospital in the Ashanti Region, Ghana. In this cross-sectional study, 58 patients visiting the hospital for routine healthcare were conveniently recruited after obtaining informed consent. Data on sociodemographic characteristics and medical history were obtained using pretested structured questionnaires. Mid-stream urine was collected for bacteria isolation and identification using standard bacterial culture and biochemical tests. Bacteria cultures ≥ 10^5^ CFU/mL were considered significant bacteriuria. The antibiotic sensitivity patterns of the bacteria isolates were evaluated using the Kirby-Bauer disc diffusion method. Bacteriuria was recorded among 15.5% (9/58) of the patients, mainly those with no previous history (77.8%) and no symptoms (55.6%) of urinary tract infections (UTIs). *E. coli* (55.6%) and *Klebsiella* spp. (44.6%) were primarily isolated from the T2DM patients. All the bacteria isolates (*E. coli* and *Klebsiella* spp.) demonstrated the highest resistance to co-trimoxazole and tetracycline (55.6%) and a complete susceptibility to amikacin and levofloxacin (100%). However, 60% of the *E. coli* isolates and 25% of the *Klebsiella* isolates were multidrug resistant (MDR; resistant to at least one antibiotic agent in three or more antimicrobial categories). The study shows that T2DM patients have bacteria in their urine which are resistant to most common antibiotics, even among those with no history of UTIs; hence, routine bacterial culture and antibiotic sensitivity testing among T2DM patients is recommended for better patient management to reduce the co-morbidities of UTIs.

## 1. Introduction

Diabetes mellitus (DM) is a heterogeneous group of metabolic conditions characterized by hyperglycaemia due to impaired insulin secretion, action, or both [1]. There are two types of DM: Type 1 DM, an autoimmune disease destroying the insulin-producing pancreatic beta cells, and Type 2 DM (T2DM), when the body does not produce enough insulin or resists it [1–3]. The liver plays a crucial role in regulating carbohydrate metabolism, storing glucose as glycogen, and synthesising glucose from non-carbohydrate sources [4]. Hence, in T2DM, the loss of a direct effect of insulin to suppress hepatic gluconeogenesis and glycogenolysis typically leads to increased hepatic glucose production [5]. Worldwide, about 422 million adults are affected with T2DM, with an estimated death rate of 1.5 million annually. Also, T2DM is projected to be the seventh leading cause of death by 2030 [6, 7]. Again, 23.6 million adults in Africa have T2DM, with a prevalence rate of 4.5%, according to the International Diabetes Federation (IDF) [8]. Previous studies showed a modest prevalence rate of less than 0.02% in the adult population in Ghana, whereas current rates range from 6.2% to 13.9% [9, 10].

Bacteriuria is defined as the presence of bacteria in the urine. Asymptomatic bacteriuria (ASB) is the presence of bacteria in the urine without urinary symptoms; a common clinical condition that often requires deciding whether to start antimicrobial treatment [11]. Symptomatic bacteriuria or urinary tract infections (UTIs) refer to the presence of bacteriuria accompanied by symptoms including polyuria, dysuria, and urgency for urination. UTIs are among the common reasons antimicrobials are prescribed for patients [12]. Yet, there are few indications to treat ASB, and untimely treatment of ASB contributes to developing antibiotic resistance. Also, treating ASB is not beneficial for most patients and may be detrimental. For example, a retrospective study of more than 2700 patients with ASB showed that antimicrobial treatment did not improve outcomes and was associated with more extended hospitalization [13].

Patients with T2DM are at increased risk of infections, with the urinary tract being the most affected [14–16]. Various impairments in the immune system, in addition to poor metabolic control of diabetes and incomplete bladder emptying due to autonomic neuropathy, contribute to the pathogenesis of UTIs in these patients [17–19]. Also, advanced age enhances the risk for UTIs among T2DM patients [20]. A prevalence of UTI of 20.6% has been reported among T2DM patients in Kisii Referral Hospital, Kenya [21]. Also, in Ghana, a UTI prevalence of 9.2% has been reported among T2DM patients in Accra [22]. These previous and other studies have reported *Escherichia coli*, *Klebsiella* spp., *Pseudomonas* spp., and *Staphylococcus aureus* as the primary uropathogens involved in UTIs among T2DM patients, mainly resistant to ciprofloxacin, ampicillin, and co-trimoxazole [21–23].

Worldwide, the spectrum of UTIs among T2DM patients ranges from ASB to cystitis, pyelonephritis, and severe urosepsis. However, serious complications of UTI, such as emphysematous cystitis and pyelonephritis, renal abscesses, and renal papillary necrosis, are also encountered more frequently in patients with T2DM than in the general population [24, 25]. Therefore, it is imperative to monitor patients with T2DM for bacteriuria to avert the future burden of severe UTIs among these patients. This study assessed the prevalence of bacteriuria and the antibiogram patterns of bacteria isolates among T2DM patients visiting the Ejisu Government Hospital in the Ashanti Region, Ghana. This study adds to the scanty data concerning bacteriuria among T2DM patients in Ghana.

## 2. Materials and Methods

### 2.1. Study Design and Site

A cross-sectional study design was employed in this research from June to August 2023 at the Ejisu Government Hospital in the Ejisu-Juaben Municipality situated in the central part of the Ashanti Region of Ghana. The municipality covers an area of approximately 637.2 square kilometres with a population of 180,723, comprising 87,836 males and 92,887 females, according to the 2021 Ghana Population and Housing Census [26]. The hospital began as a health centre in 1972 and was re-designated as a Government Hospital in 2010. It is a 64-bed health facility that serves as the leading healthcare provider to the indigenous people in the municipality and offers outpatient, obstetrical, surgical, maternal, and laboratory services.

### 2.2. Study Size Estimation

The sample size was calculated using a UTI prevalence of 9.2% among T2DM patients in Accra, Ghana [22], and the Fischer expression for cross-sectional studies. Hence, at an 80% confidence level and a 5% margin of error, the sample size was 55, as shown below. Yet, 58 patients who agreed to partake in the study were recruited by convenient sampling.

(1) $$\begin{array}{c} n=\frac{z^{2}\times p\left(1-p\right)}{m^{2}}=\frac{1.28^{2}\times 0.092\times \left(1-0.092\right)}{0.05^{2}}=54.7\approx 55, \end{array}$$

where *n* = sample size; *z* = *z*-score value (1.28) from a normal distribution at 80% confidence level; *m* = margin of error; *p* = prevalence of UTI among T2DM patients.

### 2.3. Data and Sample Collection

Data on sociodemographic characteristics and medical history of the T2DM patients who consented to partake in the study were collected using a pretested structured questionnaire. The questionnaire was validated by pilot testing and administered by the study researchers at the study site. The questions were explained to the participants in the Ghanaian Akan language. The patient's fasting blood glucose levels were assessed using the OneTouch Ultra Blood Glucose Metre (Johnson and Johnson, USA). Also, early morning mid-stream urine was collected from the patients into sterile screw-capped plastic containers and transported in a cold box to the Clinical Analysis Laboratory (CAn Lab), Kwame Nkrumah University of Science and Technology (KNUST), Ghana, for bacteria isolation and antibiotic sensitivity assessment.

### 2.4. Sample Culturing Method and Biochemical Identification of Bacteria Isolates

The urine samples were inoculated on chocolate and cystine lactose electrolyte deficient (CLED) agar plates and incubated at 37°C for 18–24 h. The chocolate agar plates were incubated in a carbon dioxide jar. The bacteria cultures ≥ 10^5^ CFU/mL were considered to have significant growth, and isolates were identified using standard bacteriological techniques such as colonial characteristics and Gram staining. Next, the bacteria isolates (Gram-negative bacilli) were speciated using biochemical tests. The biochemical characteristics of the bacteria isolates are presented in Table 1.

### 2.5. Antibiotic Sensitivity Testing

The antibiotic sensitivity testing of the bacteria isolates was performed on the Mueller–Hinton agar using the Kirby-Bauer disc diffusion method. The antibiotics evaluated were cephalexin (30 μg), tetracycline (30 μg), ciprofloxacin (5 μg), ampicillin (20 μg), amikacin (30 μg), co-trimoxazole (25 μg), chloramphenicol (30 μg), levofloxacin (5 μg), ofloxacin (5 μg), norfloxacin (10 μg), ceftriaxone (30 μg), and nitrofurantoin (10 μg). These antibiotic discs were selected based on locally used antibiotics in Ghana. The growth inhibition zones were measured using a calliper after incubation for 18 h at 37°C. The bacteria isolates were classified as sensitive, intermediate or resistant based on the growth inhibition zones. Moreover, the bacteria isolates resistant to at least one antibiotic agent in three or more antimicrobial categories were considered multidrug resistant (MDR) [27].

### 2.6. Data Analysis

The data were analyzed using the Statistical Package for Social Sciences (SPSS) software (IBM, version 23.0). The data were expressed in percentages and frequencies, and Fisher's exact test was used to determine significant differences between the variables. Statistical significance was accepted in all comparisons at a *p* value less than 0.05.

### 2.7. Ethical Considerations

Ethical approval was obtained from the Committee on Human Research, Publication, and Ethics (CHRPE) of the School of Medicine and Dentistry, KNUST (CHRPE/AP/602/23). Permission was also sought from the study site. Further, written informed consent was obtained from the T2DM patients after a full explanation of the purpose of the study was given.

## 3. Results

### 3.1. Prevalence of Bacteriuria Among the T2DM Patients

**Figure 1:**
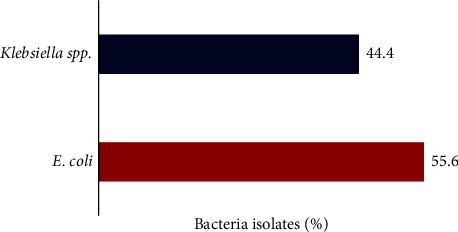
Predominant bacteria isolates from the T2DM patients.

Bacteriuria was recorded among 15.5% of the patients (9/58), predominantly *E. coli* (55.6%), as shown in Figure 1. Bacteriuria was primarily identified among patients in their fifties (3/9; 33.3%) and sixties (3/9; 33.3%), females (7/9; 77.8%) and patients with higher fasting blood glucose levels (6/9; 66.7%), although statistically insignificant. Notably, most patients with bacteriuria had no previous history (7/9; 77.8%) and no present symptoms (5/9; 55.6%) of UTIs, as presented in Table 2.

### 3.2. Antibiogram Patterns of Bacteria Isolates Among the T2DM Patients

Table 3 shows the antibiogram patterns of the individual *E. coli* isolates, whereas Table 4 shows the antibiogram patterns of the individual *Klebsiella* isolates. These bacteria isolates demonstrated the highest resistance to co-trimoxazole and tetracycline (55.6%) and a complete susceptibility to amikacin and levofloxacin. However, 60% (3/5) of the *E. coli* isolates and 25% (1/4) of the *Klebsiella* isolates were considered MDR because they were resistant to at least one antibiotic agent in three antimicrobial categories [27].

## 4. Discussion

This study revealed that 15.5% (9/58) of the patients had bacteriuria, with 55.6% asymptomatic and 44.4% presenting with symptoms; hence, the UTI prevalence in this current study among the patients was 6.9% (4/58). The prevalence of UTIs observed in this study among T2DM patients appears to diverge from findings in Southern and Eastern Ethiopia, where UTI rates ranged from 13.8% to 15.4% [28, 29], and from the National Diabetes Management and Research Centre in Ghana, where a prevalence of 28.0% was reported [10]. Nevertheless, the current UTI prevalence aligns more closely with the 9.2% UTI prevalence documented among T2DM patients at the Korle-Bu Teaching Hospital in Ghana [22]. Geographical disparities, host immune response and personal hygiene could potentially contribute to the diversity in these prevalence rates [22, 28].

Some studies have reported ASB among T2DM patients in different geographical settings. In Eastern India, an ASB prevalence of 21.3% has been reported among 80 T2DM patients, whereas 26.4% has been reported among 102 T2DM patients in the Sekondi-Takoradi Metropolis, Ghana [30, 31]. The ASB prevalence rates reported in these previous studies are higher than recorded (8.6%) among the 58 T2DM patients at the Ejisu Government Hospital in the Ashanti Region, Ghana. These disparities in the prevalence rates of ASB could be due to geographical location and sample size. Also, some studies have suggested that poor glycaemic control and long-standing diabetes are factors associated with the occurrence of ASB [32, 33]. Yet, sociodemographic characteristics and medical history did not influence the prevalence of bacteriuria in this current study, similar to a previous study in Ethiopia [34]. However, the duration of diabetes and high fasting blood glucose levels have been reported to be associated with bacteriuria in other studies [22, 23, 28, 31].

Gram-negative bacteria are mostly reported for their involvement in the cause of UTIs [10, 22, 28, 31]. *E. coli* (55.6%) and *Klebsiella* spp. (44.4%) were identified as the main bacteria in the urine samples of the T2DM patients. Compared to previous studies, Yenehun and colleagues reported *E. coli* as the most common uropathogenic bacteria (63.6%) [23]. Also, Meghana and Ravi found *E. coli* (59%) and *Klebsiella* spp. (18%) to be the most common bacteria associated with UTIs among T2DM patients [35]. In Ghana, a similar study by Owusu and colleagues reported a prevalence of 50% for *E. coli* among T2DM patients. On the contrary, a study done by Forson and colleagues reported *Klebsiella* spp. (55.6%) and *E. coli* (31.3%) as the predominant uropathogenic bacteria among T2DM patients [10, 22]. The high prevalence of *E. coli* in the urine samples might be attributed to the adherence of type 1-fimbriated *E. coli* to patients' uroepithelial cells [22, 28].

The bacteria isolates from this study demonstrated the highest resistance to co-trimoxazole and tetracycline and a complete susceptibility to amikacin and levofloxacin. Almost all the *E. coli* isolates from this study were MDR, whereas three of the four *Klebsiella* isolates were susceptible to all antibiotics except co-trimoxazole. Similar to the current research findings, MDR *E. coli* has been identified in urine samples collected from T2DM patients at Laquintinie Hospital in Douala, Cameroon. Notably, as reported in this study, the isolates exhibited resistance to ciprofloxacin, amoxicillin, and ofloxacin [36]. Similarly, MDR *E. coli* with resistance to cefuroxime, ampicillin, and gentamicin has been isolated from urine samples of T2DM patients visiting the Korle-Bu Teaching Hospital, Ghana [22]. However, it has been reported that bacteria isolated from urine samples of T2DM patients at the National Diabetes Management and Research Centre, Ghana, were susceptible to cefotaxime, cefuroxime, nitrofurantoin, and ceftriaxone [10]. Several factors contribute to uropathogenic susceptibility and antibiotic resistance, including the overuse, underuse or misuse of antibiotics and the lack of diagnostic laboratory facilities for antibiotic sensitivity testing [34]. Also, the variations in antibiotic dependence among T2DM patients across different countries may cause the observed variations in the antibiotic resistance [22]. According to Ghana's standard treatment recommendations, ciprofloxacin and ceftriaxone are to be used to treat UTIs [37]. However, this current study revealed some resistance to ciprofloxacin among the isolated *E. coli* and *Klebsiella* spp., possibly resulting from its regular usage [38, 39]. Ceftriaxone, on the other hand, was sensitive to most bacteria isolates.

## 5. Conclusion and Recommendation

The prevalence of bacteriuria among the T2DM patients visiting the Ejisu Government Hospital in the Ashanti Region of Ghana was 15.5%, with *E. coli* and *Klebsiella* spp. as the isolates. This study showed that the patients have bacteria in their urine that are resistant to the standard antibiotics used in Ghana, even among those with no history of UTIs, which is alarming. It is recommended that routine bacteria culture and antibiotic sensitivity testing be carried out for T2DM patients for better patient management to reduce the co-morbidities of UTIs. Also, it is recommended that health education on the appropriate use of antibiotics should be intensified in Ghana by the Ministry of Health to reduce the increasing trend of antibiotic resistance.

## 6. Limitation

The study was done with a smaller sample size, which could account for the lower prevalence of bacteriuria compared to other previous studies. Although the prevalence rate was low, it could be influenced by the large number of females recruited since, irrespective of their diabetes state, females are more likely to have UTIs than males because of their anatomy.

## Figures and Tables

**Table 1:** Biochemical characteristics of bacteria isolates.

Biochemical tests	*E. coli*	*Klebsiella* spp.
Urea	Negative	Positive
Indole	Positive	Negative
Simmon's citrate	Negative	Positive
Voges–Proskauer	Negative	Positive
TSI (gas production)	Positive	Positive
TSI (H_2_S production)	Negative	Negative
TSI (lactose fermentation)	Positive	Negative

Abbreviations: H_2_S, hydrogen sulphide; TSI, triple sugar iron.

**Table 2:** Prevalence of bacteriuria among the T2DM patients.

Variable	Negative [*n* (%)]	Positive [*n* (%)]	Total [*N* (%)]	*p* value
Overall	49 (84.5)	9 (15.5)	58 (100)	
Age (years)				0.976
40–49	5 (10.2)	1 (11.1)	6 (10.3)	
50–59	17 (34.7)	3 (33.3)	20 (34.5)	
60–69	16 (32.7)	3 (33.3)	19 (32.8)	
70–79	8 (16.3)	1 (11.1)	9 (15.5)	
80–89	3 (6.1)	1 (11.1)	4 (6.9)	
Gender				0.513
Female	40 (81.6)	7 (77.8)	47 (81.0)	
Male	9 (18.4)	2 (22.2)	11 (19.0)	
Marital status				0.261
Single	27 (55.1)	4 (44.4)	31 (53.4)	
Married	22 (44.9)	5 (55.6)	27 (46.6)	
Duration of diabetes (years)				0.634
< 5	27 (55.2)	4 (44.4)	31 (53.4)	
6–15	18 (36.8)	4 (44.4)	22 (37.9)	
> 15	4(8.2)	1 (11.1)	5 (8.6)	
Fasting blood glucose				0.337
< 5.6 mmol/L	10 (20.4)	3 (33.3)	13 (22.4)	
≥ 5.6 mmol/L	39 (79.6)	6 (66.7)	45 (77.6)	
Previous history of UTI?				0.902
No	41 (83.7)	7 (77.8)	48 (82.8)	
Yes	8 (16.3)	2 (22.2)	10 (17.2)	
Present symptoms of UTI?				0.661
No	16 (32.7)	5 (55.6)	21 (36.2)	
Yes	33 (67.3)	4 (44.4)	37 (63.8)	

*Note:* Statistical difference calculated by Fisher exact test.

Abbreviation: UTI, urinary tract infection.

**Table 3:** Antibiogram patterns of the *E. coli* isolates.

Antibiotics	EC01	EC02	EC03	EC04	EC05
Cephalexin	R	S	S	R	S
Tetracycline	R	R	R	S	R
Ciprofloxacin	R	S	R	R	S
Nitrofurantoin	S	S	R	S	R
Ampicillin	R	S	I	S	S
Co-trimoxazole	R	R	S	R	S
Amikacin	S	S	S	S	S
Chloramphenicol	S	S	R	I	S
Levofloxacin	S	S	S	S	S
Norfloxacin	R	S	R	R	S
Ofloxacin	R	S	R	R	S
Ceftriaxone	R	S	S	S	S
MDR	**+**	−	**+**	**+**	−

Abbreviations: EC01-EC05, *E. coli* isolates; I, intermediate; MDR, multidrug resistant; R, resistant; S, sensitive.

**Table 4:** Antibiogram patterns of the *Klebsiella* isolates.

Antibiotics	KS01	KS02	KS03	KS04
Cephalexin	S	R	S	S
Tetracycline	S	R	S	S
Ciprofloxacin	S	R	S	S
Nitrofurantoin	S	S	S	S
Ampicillin	S	S	S	S
Co-trimoxazole	S	R	S	R
Amikacin	S	S	S	S
Chloramphenicol	S	I	S	S
Levofloxacin	S	S	S	S
Norfloxacin	S	R	S	S
Ofloxacin	S	R	S	S
Ceftriaxone	S	R	S	S
MDR	−	+	−	−

Abbreviations: I, intermediate; KS01-KS04, *Klebsiella* spp.; MDR, multidrug resistant; R, resistant; S, sensitive.

## Data Availability

All the data obtained and analyzed are included in this manuscript.
